# Implication of proliferation gene biomarkers in pulmonary hypertension

**DOI:** 10.1002/ame2.12191

**Published:** 2021-11-22

**Authors:** Yi Yan, Rong Jiang, Ping Yuan, Li Wen, Xiao‐Bin Pang, Zhi‐Cheng Jing, Yang‐Yang He, Zhi‐Yan Han

**Affiliations:** ^1^ Institute for Cardiovascular Prevention (IPEK) Ludwig‐Maximilians‐University Munich Munich Germany; ^2^ DZHK (German Centre for Cardiovascular Research) partner site Munich Heart Alliance Munich Germany; ^3^ Department of Cardio‐Pulmonary Circulation Shanghai Pulmonary Hospital, Tongji University School of Medicine Shanghai China; ^4^ Department of Cardiology The First Affiliated Hospital, Chongqing Medical University Chongqing China; ^5^ School of Pharmacy Henan University Henan China; ^6^ State Key Laboratory of Complex, Severe, and Rare Diseases, and Department of Cardiology Peking Union Medical College Hospital, Chinese Academy of Medical Sciences and Peking Union Medical College Beijing China; ^7^ State Key Laboratory of Cardiovascular Disease and FuWai Hospital Chinese Academy of Medical Sciences and Peking Union Medical College Beijing China

**Keywords:** metabolism associated genes, metabolomics, proliferation, pulmonary hypertension, transcriptomics

## Abstract

**Objective/Background:**

Proliferation is a widely recognized trigger for pulmonary hypertension (PH), a life‐threatening, progressive disorder of pulmonary blood vessels. This study was aimed to identify some proliferation associated genes/targets for better comprehension of PH pathogenesis.

**Methods:**

Human pulmonary arterial smooth muscle cells (hPASMCs) were cultured in the presence or absence of human recombinant platelet derived growth factor (rhPDGF)‐BB. Cells were collected for metabolomics or transcriptomics study. Gene profiling of lungs of PH rats after hypoxia exposure or of PH patients were retrieved from GEO database.

**Results:**

90 metabolites (VIP score >1, fold change >2 or <0.5 and *p* < .05) and 2701 unique metabolism associated genes (MAGs) were identified in rhPDGF‐BB treated hPASMCs compared to control cells. In addition, 1151 differentially expressed genes (313 upregulated and 838 downregulated) were identified in rhPDGF‐BB treated hPASMCs compared to control cells (fold change >2 or <0.5 and *p* < .05). 152 differentially expressed MAGs were then determined, out of which 9 hub genes (IL6, CXCL8, CCL2, CXCR4, CCND1, PLAUR, PLAU, HBEGF and F3) were defined as core proliferation associated hub genes in protein proten interaction analysis. In addition, the hub gene‐based LASSO model can predict the occurrence of PH (AUC = 0.88). The expression of CXCR4, as one of the hub genes, was positively correlated to immune cell infiltrates.

**Conclusion:**

Our findings revealed some key proliferation associated genes in PH, which provide the crucial information concerning complex metabolic reprogramming and inflammatory modulation in response to proliferation signals and might offer therapeutic gains for PH.

## Graphical abstract

Proliferation associated hub genes in pulmonary arterial smooth muscle cells (PASMCs) in response to platelet derived growth factor‐BB (PDGF‐BB) were explored by virtue of transcriptomics and metabolomics. Those hub genes might be responsible for proliferating PASMCs phenotype and associated with immune cell infiltrates into the lung tissues, contributing to the development of pulmonary vascular remodeling (created with BioRender.com).

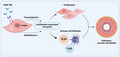

## 1 INTRODUCTION

Pulmonary hypertension (PH) is a vicious cardio‐pulmonary disorder and manifested by progressive increase in pulmonary artery pressure and pulmonary vascular resistance, which ultimately leads to right heart failure and even death.^1^ Multiple factors including genetic predisposing genes,^2^, ^3^ epigenetic modulations,^4^, ^5^ inflammation,^6^ altered metabolism^7^, ^8^ and environment insults such as hypoxia^9^ are reported to cause the remodeling of the pulmonary vasculature, as manifested by overproliferation, anti‐apoptosis and high migratory capability of vascular cells.^10^ According to WHO classification, PH is categorized into 5 main groups, with pulmonary arterial hypertension (PAH) to be Group 1 PH.^11^ As of today, most of therapies are targeted against PAH, including endothelin receptor antagonists, phosphodiesterase type 5 inhibitors, and prostacyclin analogues.^12^ These therapies help to improve exercise capacity, hemodynamics and quality of life. However, none of the current treatments are actually curative and long‐term prognosis still remains poor.

As proliferation is a critical trigger for PH development, the factors/elements in regulation of proliferation has emerging as a research focus against pulmonary vascular remodeling. For example, low‐dose FK506 reverses PH in rats with medial hypertrophy following monocrotaline and neointima formation of Sugen5416/Hypoxia PH rats (a severe PH model) via rescue of bone morphogenetic protein receptor‐2 (BMPR2) signaling dysfunction,^13^ which is widely recognized in circulating system and lung tissues of PH patients.^14^, ^15^ At the mechanism level, the induction of BMPR2 signaling by FK506 is not only associated with the reversal of endothelial cells (ECs) dysfunction, but also with the inhibition of pulmonary arterial smooth muscle cells (PASMCs) proliferation, as evident by a direct effect of FK506 in inhibiting platelet derived growth factor (PDGF) induced proliferation. PDGF is regarded as the most potent mitogen for PASMCs^16^ and the subtype PDGF‐BB (dimeric form of PDGF‐B) is widely applied as a stimuli for PASMCs functional study in PH. Moreover, a higher PDGF‐B and its receptor PDGFR‐beta mRNA expression was observed in small pulmonary arteries from patients with idiopathic PAH, and both PDGF‐B and PDGFR‐beta were localized in PASMCs in small remodeled pulmonary arteries.^17^ The elevated PDGF signaling contributes to the pathobiology of PAH^18^ by regulation of proliferation and migration of pulmonary vascular SMCs, and its inhibition abolishes pulmonary vascular remodeling in two PH models.^19^ Therefore, a better understanding into PDGF‐BB mediated gene/metabolite profiling would aid in the discovery of novel target to reverse vascular remodeling. Systemic or local inflammation is another feature of PH.^20^, ^21^ The proliferation associated alteration in lung tissues and its impact on immune cell infiltration would also shed some light on the pathogenesis of PH.

Here, we sought to scrutinize the gene/metabolite alterations in PASMCs in response to PDGF‐BB, identify some proliferation associated genes/targets by virtue of metabolomics and transcriptomics and unveil the potential link between proliferation and inflammation, which might provide new approaches for the treatment of PH.

## 2 METHODS

### 2.1 Cell culture and sample collection

Human pulmonary artery smooth muscle cells (hPASMCs) were purchased from the American Type Culture Collection (ATCC; Cat#. PCS‐100‐023) and cultured at 37°C in the incubator containing 5% CO_2_ in Dulbecco's Modified Eagle Medium: Nutrient Mixture F‐12 (DMEM/F‐12) (Gibco; Cat#. 11320033) containing 10% (v/v) fetal bovine serum (FBS) (Gibco; Cat#.10437‐028), 100 μg/ml streptomycin and 100 U/ml penicillin (Gibco; Cat#. 15140‐122).

For metabolomic study, hPASMCs were seeded into 6‐well plates at a density of 0.5 × 10^6^/ml. Twenty‐four hours later cells were starved with DMEM/F‐12 containing 0.5% fetal bovine serum for another 24 h. Cells were gently washed with PBS followed by addition of fresh blank medium with recombinant human PDGF‐BB (rhPDGF‐BB) (R& D systems; Cat#. 220‐BB) at the concentration of 20 ng/ml or vehicle for 24 h (*n* = 6/group). Cells were collected, washed, centrifuged at 300 *g* at 4°C for 10 min and resuspended with PBS to reach a final density of 1 × 10^5^/ml. One milliliter cell suspension per sample was centrifugated at 300 *g* at 4°C for 5 min. Supernatants were discarded and cell pellets were stored at −80°C. Freezing and thawing cycle was avoided before sample processing in case of potential degradation of metabolites.

For bulk RNA sequencing, hPASMCs were seeded into 6‐well plates and starved as aforementioned. Cells were gently washed with PBS followed by addition of fresh blank medium with recombinant human PDGF‐BB (rhPDGF‐BB) (R& D systems; Cat#. 220‐BB) at the concentration of 20 ng/ml or vehicle BB for 6 h (*n* = 3/group) before collection. Cells were then washed, centrifuged at 300 *g* at 4°C for 10 min. Supernatants were discarded and cell pellets were stored at −80°C before further use.

### 2.2 LC/MS analysis and data process

One milliliter of MeOH:ACN:H_2_O (2:2:1, v/v) solvent mixture was added into the cell samples followed by sonification. Cells were prepared as previously described.^7^ Liquid chromatographic separation for processed samples was achieved on a ZORBAX Eclipse Plus C18 column (2.1 × 100 mm, 3.5 μm, Agilent, USA) maintained at 45°C, whereas mass spectrometry was performed on a Nexera X2 system (Shimadzu, Japan) coupled with a Triple TOF 5600 quadrupole‐time‐of‐flight mass spectrometer (AB SCIEX, USA). All the steps for LC/MS analysis and data preprocessing were depicted in our previous study.^7^

We used partial least squares discriminant analysis (PLS‐DA) to distinguish the overall difference in metabolic profile between rhPDGF‐BB treated‐ and control hPASMCs. Variables with a variable weight value (Variable Important in Projection, VIP) >1 were considered to be distinguishing among groups. The enriched metabolic pathways of metabolites with VIP >1 and fold change (FC) >2 or <0.5 and corresponding *p* < .05 between rhPDGF‐BB treated‐ versus vehicle treated hPASMCs were further analyzed by Metaboanalyst^22^ (v5.0, https://www.metaboanalyst.ca). The top 10 enriched metabolite pathways were selected as key word for analysis in Genecards^23^ (https://www.genecards.org). Genes with relevance score >8 were defined as metabolism associated genes (MAGs).

### 2.3 RNA isolation, RNA sequencing and analysis

Total mRNA was isolated from frozen hPASMCs with Trizol (Invitrogen). RNA libraries were constructed for sequencing on the BGISEQ‐500 sequencing platform (BGI, Wuhan, China) with a single‐end read length of 50 bp. Briefly, high‐quality reads were aligned to the human reference genome (GRCh38) using HISAT2 (version 2.0.4) and Bowtie2 (version 2.2.5). Normalized gene expression was calculated using the fragments per kilobase per million mapped reads (FPKM). Student's *t* test was used for differentially expressed genes (DEGs) analysis. DEGs were defined as fold change (FC) of gene expression >2 or <0.5 and corresponding *p* < .05 between rhPDGF‐BB treated‐ versus vehicle treated hPASMCs. Principle component analysis (PCA) was adopted to distinguish the overall difference in expression gene profiling between groups. The enriched pathways of DEGs were identified by functional annotation tool Metascape^24^ (https://metascape.org/gp/index.html#/main/step1) and visualized in bar plot with barplot function in R.

### 2.4 Identification and validation of hub genes

The intersection of MAGs and DEGs in rhPDGF‐BB treated‐ versus vehicle treated cells was regarded as critical proliferation associated genes. Next, protein‐protein interaction of those genes were analyzed by STRING (v11.0, https://string‐db.org).^25^ CytoHubba plugin with MCC algorithm or MCODE plugin in Cytoscape (v 3.8.2)^26^ were used to find the hub genes of the network identified by STRING.

Hub genes were validated in human lungs from the dataset GSE117261 ^27^ with 25 control subjects and 58 PH patients and in rat lungs from the dataset GSE85618 ^28^ including 4 PH rats after hypoxia exposure and 4 rats in normoxic condition. All the datasets were obtained from Gene Expression Omnibus (GEO) database.

### 2.5 Construction of LASSO regression model

Least Absolute Shrinkage and Selection Operator (LASSO) regression model was constructed with the hub gene panel by glmnet package in R to distinguish PH patients from control subjects. A model index for each sample was generated using the regression coefficients to weight the expression of each hub gene. Samples of dataset GSE117261 were randomly assigned to training set (70%) and test set (30%). ROC curves were generated to evaluate the ability of LASSO model to identify PH by ROCR package.

### 2.6 Immune cell infiltrates and its relationship with CXCR4

To decipher the immune cell heterogeneity of human lung tissues, cell type enrichment analysis from gene expression data of GSE117261 for different immune cells was performed based on webtool xCell^29^ (https://xcell.ucsf.edu). The relationship of CXCR4 with different cell infiltrates were examined by Pearson correlation analysis. *p < .05* represents significant correlation.

### 2.7 Data visualization and statistics

Volcano plot was generated to display the overview of distinguishing metabolites or genes between rhPDGF‐BB treated‐ and vehicle treated hPASMCs. The expression of the distinct metabolites or indicated genes in each individual sample was plotted in heatmap. Hub gene network was visualized by Cytoscape. Correlation of immune cell infiltrates with CXCR4 expression was plotted in scatterplot in R.

Data are presented as the mean ± standard error of the mean (SEM). Statistical differences between two groups were evaluated with 2‐tailed unpaired *t* test if the samples were normally distributed. Otherwise, Mann‐Whitney test was used to detect the difference (GraphPad Prism 8). *p < .05* denotes significant differences between two groups.

## 3 RESULTS

### 3.1 Altered metabolic signature in hPASMCs in response to rhPDGF‐BB and identification of MAGs

**FIGURE 1:**
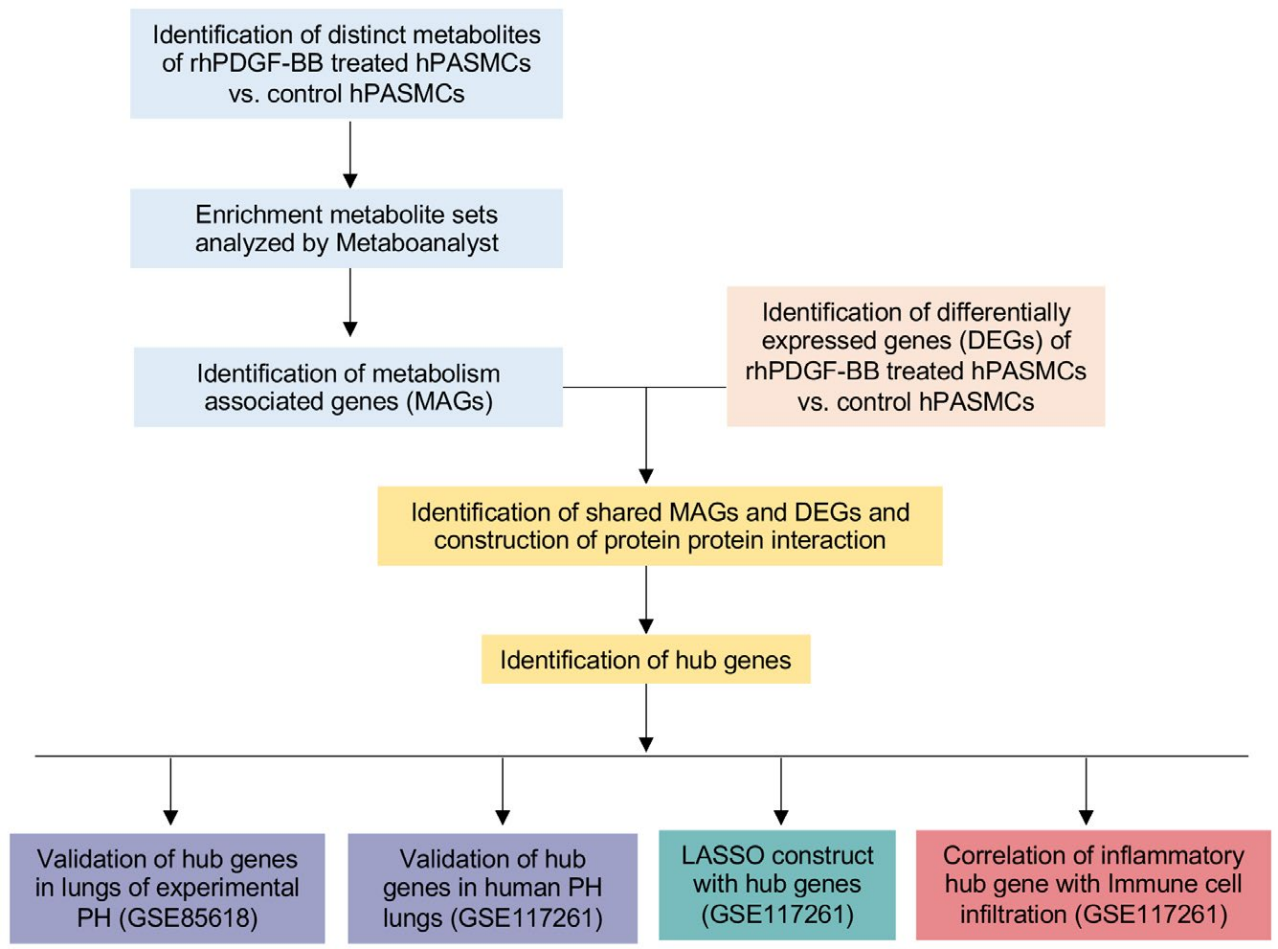
Main analysis flowchart. Identification of distinguishing metabolites of recombinant human recombinant PDGF‐BB (rhPDGF‐BB) treated human pulmonary arterial smooth muscle cells (hPASMCs) versus vehicle treated PASMCs. Enrichment of metabolite sets were analyzed by Metaboanalyst. Top 10 enriched pathways were identified and selected for further identification of metabolism associated genes (MAGs). Next, the shared MAGs and differentially expressed genes (DEGs) in rhPDGF‐BB treated hPASMCs versus control cells were identified. Protein protein interaction were constructed. Hub gene were identified by cytoHubba or MCODE add‐ins in Cytoscape. Common hub genes were validated in lungs of PH rats after hypoxia exposure from dataset GSE85618 and in lungs of PH patients from dataset GSE117261. LASSO regression model was constructed based on hub genes for PH prediction. The correlation of immune cell infiltration in lung tissues of dataset GSE117261 with inflammatory hub gene was also investigated

**FIGURE 2:**
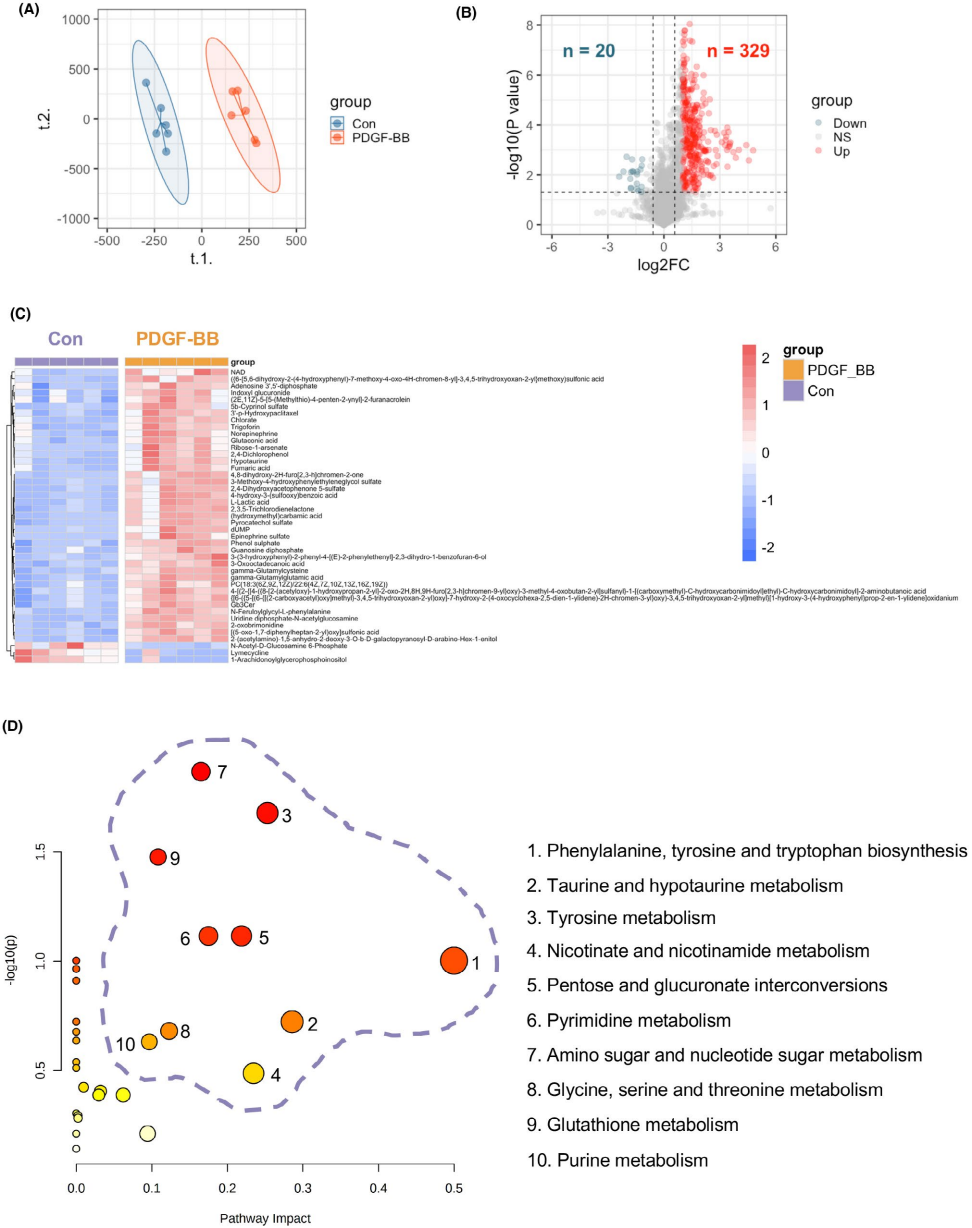
Identification of metabolites in response to rhPDGF‐BB treatment and enriched pathways of distinguishing metabolites in hPASMCs. (A) Partial least squares‐discriminant analysis (PLS‐DA) demonstrated a well separated sample distribution of rhPDGF‐BB treated hPASMCs (PDGF‐BB) and control hPASMCs (Con) and visualized in scatter plot (*n* = 6). (B) 329 upregulated metabolites (red dots) and 20 down‐regulated metabolites (dark green) were identified and visualized in volcano plot (Fold change >2 or <0.5 and *p* < .05). (C) Expression of distinguishing metabolites including top 40 upregulated and 3 downregulated metabolites with VIP score >1 were visualized in heatmap. (D) Enriched pathways of all distinguishing metabolites (Fold change >2 or <0.5, *p* < .05 and VIP score >1) were identified in Metaboanalyst

As illustrated in flow chart of Figure 1, the metabolite profiles in rhPDGF‐BB treated hPASMCs and control hPASMCs were investigated. The hPASMCs in the presence of rhPDGF‐BB separated well from control cells according to PLS‐DA analysis (Figure 2A) and had a higher proliferation capacity compared to that of control cells (Figure S1). 329 up‐regulated and 20 down‐regulated metabolites were unveiled in rhPDGF‐BB treated hPASMCs compared to that of vehicle treated cells (FC >2 or <0.5, *p* < .05) (Figure 2B). Among those changed metabolites, 90 metabolites (87 were upregulated and 3 were downregulated) had a VIP score >1 and their expressions in each sample were displayed in Figure S2. Expression of distinguishing metabolites including top 40 upregulated and 3 downregulated metabolites with VIP score >1 were visualized in Figure 2C. Next, 90 metabolites distinguishing rhPDGF‐BB treated‐ and control hPASMCs were enriched in metabolite sets such as phenylalanine, tyrosine and tryptophan biosynthesis, taurine and hypotaurine metabolism, tyrosine metabolism etc., with the top 10 documented in Figure 2D. The top 10 metabolite sets were then selected for further analysis in Genecards. According to the results, there were 2701 unique genes identified as MAGs with a relevance score >8.

### 3.2 Identification of differentially expressed MAGs in response to rhPDGF‐BB treatment

**FIGURE 3:**
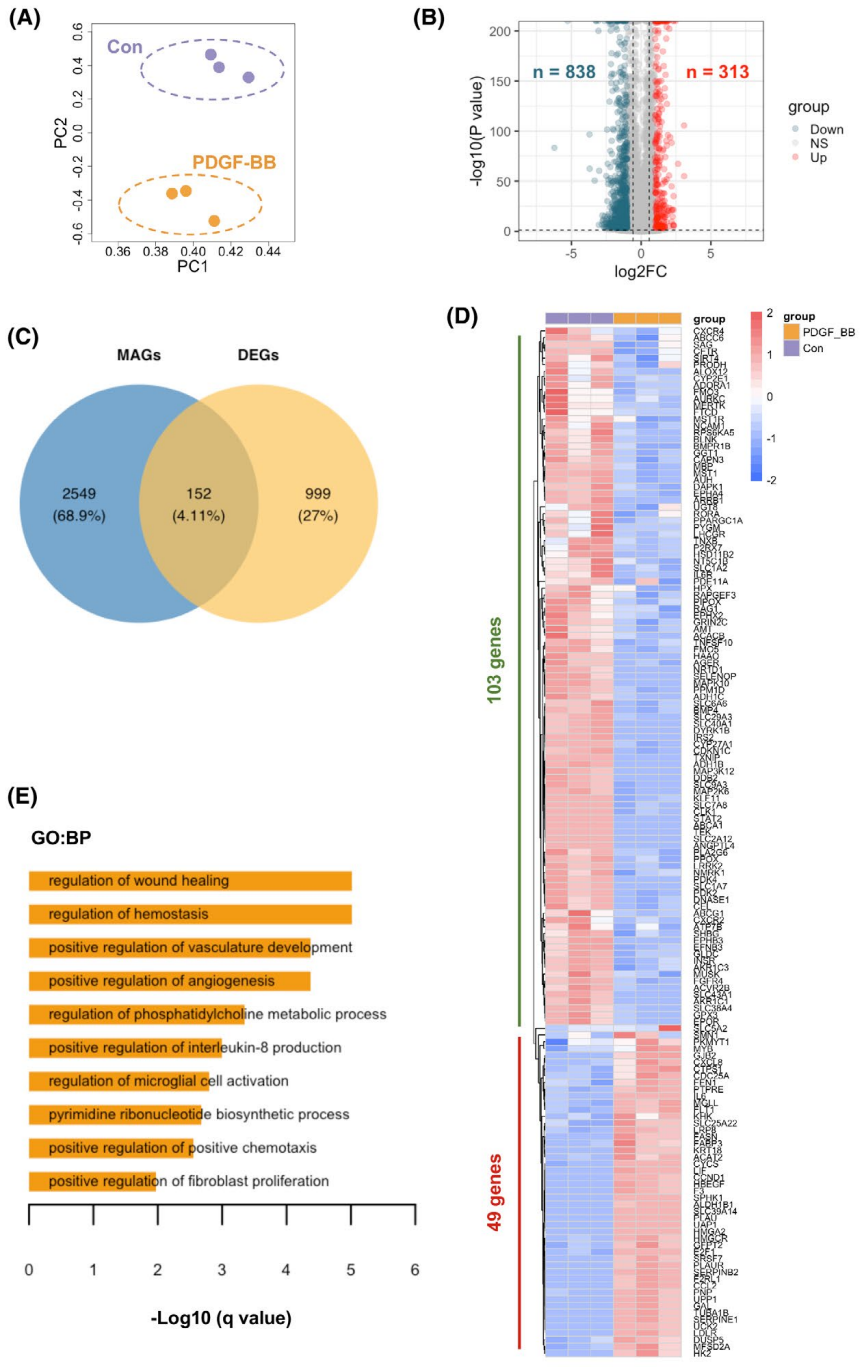
Identification of metabolites associated DEGs in response to rhPDGF‐BB treatment and enriched pathways in hPASMCs. (A) Principal component analysis (PCA) demonstrated a well separated sample distribution of rhPDGF‐BB treated hPASMCs (PDGF‐BB) and control hPASMCs (Con) (*n* = 3 per condition). (B) 313 upregulated genes (red dots) and 838 down‐regulated metabolites (dark green) were identified and visualized in volcano plot (Fold change >2 or <0.5 and *p* < .05). (C) 152 overlapped metabolites associated genes (MAGs) and DEGs between PDGF‐BB group and Con group were visualized in Venn diagram. (D) Expression of 152 overlapped genes (49 upregulated and 103 downregulated genes) were visualized in heatmap in rhPDGF‐BB treated hPASMCs and control hPASMCs. (E) The enriched GO ontology (biological processes) of 49 upregulated genes were identified by functional annotation tool Metascape and visualized in bar plot

To further narrow down the panel of MAGs in response to rhPDGF‐BB treatment, we first determined the DEGs in rhPDGF‐BB treated hPASMCs compared to control cells. The rhPDGF‐BB treated hPASMCs distributed distinctly from control cells according to PCA analysis (Figure 3A). Compared to control cells, there were 313 up‐regulated and 838 down‐regulated genes displayed in rhPDGF‐BB treated hPASMCs in Figure 3B. The Venn diagram showed 152 shared genes between DEGs and MAGs (Figure 3C). Of the 152 differently expressed MAGs, 49 were increased and 103 decreased in hPASMCs after rhPDGF‐BB treatment. The expression of those genes in hPASMCs with or without rhPDGF‐BB were shown in heatmap (Figure 3D). Next, the enriched pathways of upregulated 49 MAGs were examined by functional annotation tool Metascape. It revealed that pathways including regulation of wound healing, positive regulation of vasculature development, positive regulation of angiogenesis, positive regulation of fibroblast proliferation etc. were enriched in response to rhPDGF‐BB treatment as shown in Figure 3E.

### 3.3 Identification of rhPDGF‐BB induced proliferation associated hub genes and validation in lungs of human and experimental PH

**FIGURE 4:**
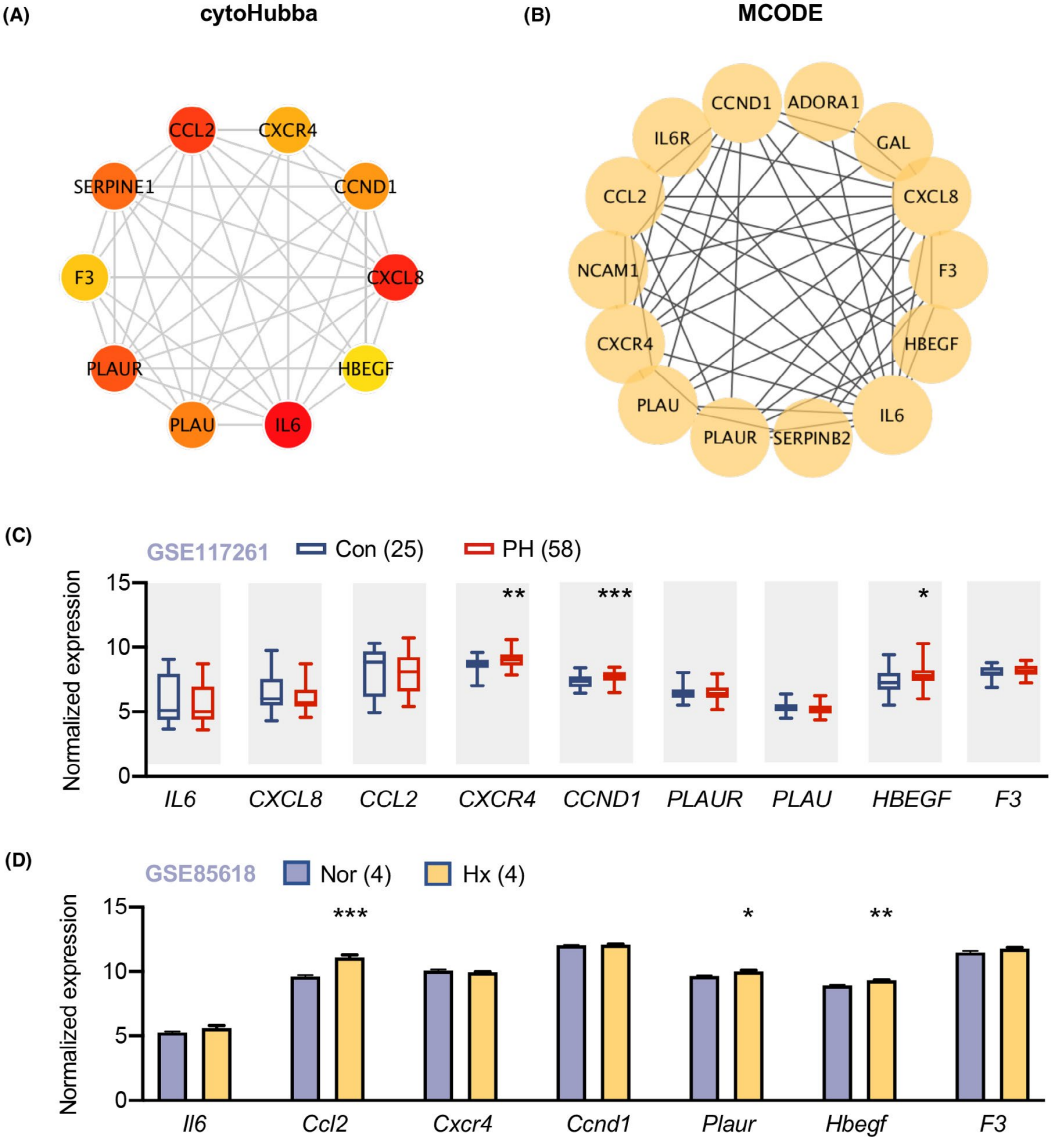
Identification of proliferation associated hub genes and validation in lungs of PH patients and PH rat models. (A) Identification of 10 hub genes by cytoHubba in Cytoscape; each circle represents unique gene and the redder the color is, the higher the MCC score is. (B) Identification of 14 hub genes by MCODE in Cytoscape; each circle represents unique gene. (C) Expression of 9 shared hub genes in lungs of 58 patients with pulmonary hypertension (PH) and 25 control subjects from GSE117261 were visualized in box plot. Data represent mean ± SEM. *p < .05, **p < .01, ***p < .001 compared to corresponding control subjects, as analyzed by unpaired *t* test. (D) The expression of hub genes in lungs of rats under hypoxia for two weeks or in normoxic condition (*n* = 4 per group). Data represent mean ± SEM. *p < .05, **p < .01, ***p < .001 compared to control rats, as analyzed by unpaired *t* test

A total of 152 genes in Figure 3D were then potentially considered as rhPDGF‐BB induced proliferation associated MAGs. 411 Protein‐protein interactions among the 152 genes were identified and visualized by STRING (Figure S3). Ten hub genes were identified by cytoHubba plugin (Figure 4A) and fourteen hub genes were revealed by MCODE plugin (Figure 4B). In particular, there were nine core hub genes (IL6, CXCL8, CCL2, CXCR4, CCND1, PLAUR, PLAU, HBEGF and F3) identified by both methods. The expressions of nine hub genes were then scrutinized in lungs of PH patients from GSE117261 dataset (Figure 4C). CXCR4 encoding C‐X‐C motif chemokine receptor 4, CCND1 encoding cyclin D1 and HBEGF encoding heparin binding EGF like growth factor (HB‐EGF) were significantly increased in PH patients (all *p* < .05). In addition, Ccl2 encoding C‐C motif chemokine ligand 2, Plaur encoding urokinase‐type plasminogen activator (u‐PA) receptor and Hbegf encoding HB‐EGF were elevated in lung tissues of PH rats after hypoxia exposure for 2 weeks compared to that at ambient atmosphere from GSE85618 dataset (Figure 4D).

### 3.4 Hub genes‐based regression model for PH prediction

**FIGURE 5:**
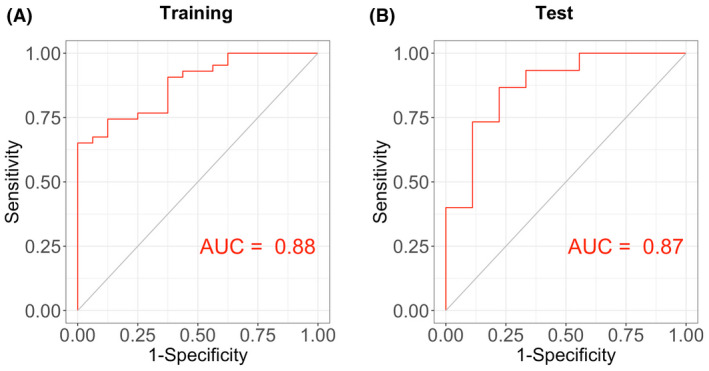
Construction of hub gene‐based LASSO regression model and its prediction for PH. (A) Receiver operating characteristic (ROC) curve analysis of training set (GSE117261) using nine hub genes. (B) ROC curve analysis of test set (GSE117261) based on nine hub genes. AUC represents area under curve

In a bid to predict PH, we then sought to construct LASSO regression model based on hub genes. There were 3 genes with non‐zero regression coefficients, and the lambda.min equalled to 0.084. We then generated the hub gene‐based model index as follows: index = CCND1 * 0.205 + CXCR4 * 0.138 + HBEGF * (0.022) − 2.253. According to ROC curve, the area under the curve (AUC) of the model was 0.88 in the training set in Figure 5A and 0.87 in the test set (Figure 5B) from GSE117261 dataset, indicative of a predictor of PH with the established LASSO regression model.

### 3.5 The correlation of Immune cell infiltration with CXCR4 expression

**FIGURE 6:**
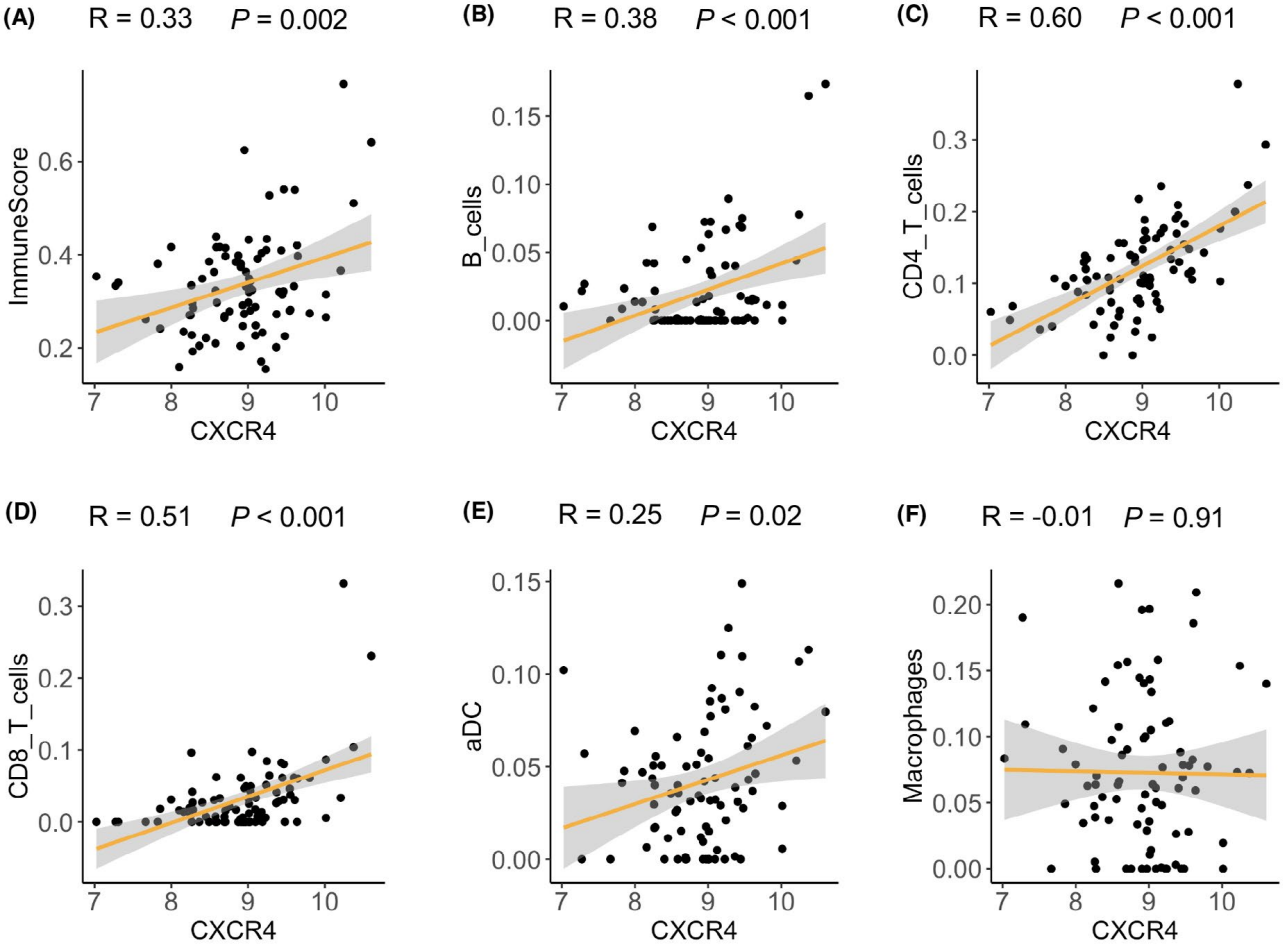
Correlation of immune cell infiltration and CXCR4 expression in human lung tissues. (A–F) CXCR4 expression in lung tissues of dataset GSE117261 was positively correlated with (A) ImmuneScore; (B) B cell infiltrates; (C) CD4^+^ T cell infiltrates; (D) CD8^+^ T cell infiltrates; (E) activated dendritic cell infiltrates and (F) macrophage infiltrates

As inflammation is considered as a critical trigger of PH development,^6^ we then investigated the association of different inflammatory cell recruitment in human lung tissues with the expression of the inflammation associated hub gene with non‐zero regression coefficient (ie. CXCR4), such that we might get a novel insight into the potential role of CXCR4 in the modulation of specific immune cell type. We found that the expression of CXCR4 was positively correlated to immune cell infiltrates as evidenced by a higher ImmuneScore in human lung tissues with higher CXCR4 levels (*r* = .33, *p* = .002) (Figure 6A). There was also a positive correlation of CXCR4 level with B cells (*r* = .38), CD4^+^ T cells (*r* = .60), CD8^+^ T cells (*r* = .51) and activated dendritic cells (*r* = .25) infiltrates, respectively (all *p* < .05), while no correlation of CXCR4 and macrophages infiltration was observed (Figure 6B–F).

## 4 DISCUSSION

In this study, the altered metabolite profiles were revealed by metabolomics and MAGs based on enriched metabolite pathways in hPASMCs were identified in response to rhPDGF‐BB treatment. In combination of our transcriptomics data, differentially expressed MAGs (ie. main proliferation associated genes) were figured out in rhPDGF‐BB treated hPASMCs compared to that of control cells, suggesting a crucial link of metabolic adaptations and proliferative phenotype in hPASMCs. We also demonstrated nine hub genes and examined their expressions in lungs of PH patients or hypoxia induced PH rats relative to corresponding controls. Moreover, the hub genes could be predictors for the occurrence of PH, which shed some insight into the development of pulmonary vascular remodeling. In addition, the CXCR4 expression was positively correlated to infiltration of multi‐immune cell types in human lung tissues. All these findings implicate a complex metabolic reprogramming and inflammatory modulation in response to proliferation signals, thus mediating the development of PH.

Multi‐omics is an emerging strategy that holds promise to discover biomarkers for risk stratification or prognosis and to identify novel therapeutic targets/pathological mechanism in diseased state. For example, metabolomics is now widely used to discover metabolic perturbations or rapid metabolic alteration in response to PH treatment/surgery. It was reported that the plasma metabolic signature of chronic thromboembolic pulmonary hypertension (CTEPH) in pre‐pulmonary endarterectomy (PEA) surgery was distinguishing from that of post‐PEA surgery.^30^ Metabolites in respond to PEA surgery could serve as suitable noninvasive markers for the evaluation on the therapeutic interventions in the future. In terms of the application of transcriptomics to PH, one of the studies showed that whole blood RNA signature in patients with PAH were distinct from that of control subjects, which was also associated with disease severity and allowed for the identification of patients with poor prognosis.^31^ This study provides important markers for risk stratification and prognosis prediction of PH. In addition, the NIH/NHLBI launched an initiative^32^ called “Redefining Pulmonary Hypertension through Pulmonary Vascular Disease Phenomics (PVDOMICS)” in 2017, which aims to foster the comprehension in PH based on biological features by virtue of “omics” analysis. This would bring great benefits to the discovery of novel targets and development of more efficacious, precision approaches to individual therapy. In our previous study, we identified spermine as the most dramatically altered metabolite in our PAH cohort by metabolomics and showed that spermine promoted the PASMCs proliferation/migration possibly via the modulation of Erk signaling by transcriptomics analysis.^7^ This finding based on multi‐omics in our previous study provides the new concept that spermine synthase might be a therapeutic target for PAH. Hence, the combination of transcriptomics and metabolomics of hPASMCs in response to the potent mitogen PDGF‐BB in the present study would permit the possible discovery of crucial proliferation associated gene markers relevant to the development of PH.

A total of nine proliferation associated hub genes were identified, some of which were cytokine/chemokine related genes. Although the hub gene IL6 didn't differ PH patients or PH rats from their corresponding controls in our selected datasets, lung‐specific IL‐6 transgenic mice develop spontaneous PH in normoxia and exhibited exaggerated hypoxia‐induced PH.^33^ In addition, IL‐6 blockade mitigated hypoxia‐induced PH and repressed the recruitment of Th17 cells and M2 macrophages in lung tissues after hypoxia exposure.^34^ These findings implicate IL6 as a trigger in pathogenesis of PH. CXCL8 (also named IL8) encoding interleukin‐8 was reported to be higher in serum of PAH patients and had a negative correlation with cardiac index. Kaplan‐Meier analysis showed that levels of interleukin‐8 predicted survival in PAH patients, with 5‐year survival of PAH patients (interleukin‐8 levels of >30 pg/ml) to be 32% compared with 58% for patients with levels ≤30 pg/ml.^35^ Nevertheless, the specific role of CXCL8 in the vasculature homeostasis remains elusive. Another inflammatory hub gene, CCL2, was found to be higher in plasma and lung tissues of IPAH patients in previous study by Sanchez et al.^36^ although no difference in CCL2 between PH patients and controls were found in dataset GSE117261. The divergency might lie in the heterogeneity of PH cohorts among two studies. In their study, PASMCs from PAH patients exhibited stronger migration and proliferation in response to CCL2. This finding suggests a critical role of CCL2 mediated PASMC biological activity in vasculature. In Consistent with the higher CXCR4 expression in lungs of PH patients in our study, CXCR4 was also much higher in lungs of PH rat model.^37^ Of note, SMC specific loss of CXCR4 inhibits SMC proliferation and retards the hypoxia‐induced PH.^38^ This would suggest a detrimental role of CXCR4 in pulmonary vascular remodeling. Intriguingly, CXCR4 expression was positively correlated with B cells‐, T cell‐ (including both CD4^+^ T and CD8^+^ T) and activated dendritic cell infiltration in human lung tissues. However, the mechanism by which CXCR4 mediates various immune cell recruitment into lung tissues in the progression of PH remains poorly understood and warrants further investigation.

For other non‐cytokine/chemokine related genes, their roles in maintenance of vascular function have also drawn great attention in PH. There are lines of evidence on the role of CCND1 encoding cyclin D1 in PASMCs and pulmonary vascular remodeling, especially in Group 3 PH (PH associated with hypoxia and lung disease). Zeng et al. showed that CCND1 silencing could attenuate the PASMC proliferation and cigarette smoke‐induced as well as monocrotaline‐induced pulmonary vascular remodeling in rats.^39^, ^40^ Moreover, noncoding RNA hsa_circ_0016070 was associated with vascular remodeling in PAH by facilitating the PASMCs proliferation via modulation of miR‐942/CCND1 axis. The miR‐942 level in lung tissues of COPD(+) PH(+) patients (Group 3 PH) was much lower than that in the COPD patients without PH, while the expression of its target genes CCND1 in the Group 3 PH was much higher compared to that of COPD controls.^41^ As of today, the PAH targeted therapy against Group 3 PH is not available, the knowledge of the mechanism by which CCND1 mediated PASMCs and other vascular cells if possible might offer new opportunity for the treatment of Group 3 PH. PLAU encodes u‐PA and PLAUR encodes its receptor u‐PA receptor. The deficiency of u‐PA dramatically reduced the right ventricular pressure after hypoxia exposure to a comparable level of wild type littermates at normoxic condition, while the loss of u‐PA receptor could only partially rescue the increase of right ventricular pressure induced by hypoxia exposure.^42^ According to a recent study by Mirna et al., plasma soluble u‐PA receptor was elevated in PH patients and primarily associated with PH due to left sided heart disease.^43^ However, how u‐PA and its receptor orchestrate the vasculature in PH development remains elucidated and is worth further investigation. F3 (also named TF) encodes tissue factor, coagulation factor III. Previous study showed that higher TF antigen, and TF mRNA in monocytes were displayed in chronic thromboembolic pulmonary hypertension (CTEPH) patients compared with control subjects. TF was also correlated with inflammatory indicators like CRP, TNF‐α and CCL2.^44^ Similar to the clinical observation, TF mRNA expression had a positive correlation with media hypertrophy (ratio of vessel wall area to total area) and mean pulmonary arterial pressure in rat model of CTEPH.^45^ The scrutiny of inflammation‐coagulation‐thrombosis cycle might open new avenue for the treatment of CTEPH. HB‐EGF encoded by HBEGF was identified as a mitogen for SMC decades ago and could be induced by multi‐mitogens like PDGF.^46^ This is in line with our observation. Besides its role in PASMCs, vascular ECs are able to express HBEGF induced by tumor necrosis factor‐α.^47^ These findings would suggest HBEGF may serve as a crucial proliferative signal amplifier and bridge the crosstalk between EC and SMCs in vessels.

Intriguingly, many of the identified hub genes (i.e., IL6, CXCL8, CXCR4, CCND1, PLAUR, PLAU) are hypoxia‐inducible factor‐alpha (HIF‐α, transcription factor modulating adaptive responses to hypoxia) target genes. HIF‐1α and HIF‐2α are regarded as the main type of HIF‐α, an important factor triggering PH development. This implicates a common molecular alteration between PDGF‐BB induced and hypoxia induced proliferative phenotype in PASMCs. To support this notion, a previous study showed PDGF promoted the metabolic shift toward glycolysis in PASMCs via activation of the PI3K/AKT/mTOR/HIF‐1α signaling.^48^ This is also in line with another study demonstrating that HIF1α upregulated genes were enriched in glycolysis and NADH regeneration.^49^ In addition, cytoskeletal protein paxillin tyrosine phosphorylation were elevated in pulmonary vasculature of hypoxia‐induced PH mice, which was abolished by PDGF‐BB antagonist (imatinib). In the same study, the increase of paxillin tyrosine phosphorylation in human PASMC was blocked by HIF‐1α depletion or by imatinib, suggesting the augmentation/phosphorylation of paxillin in PASMC responded to PDGF‐BB could be at least partially regulated by HIF‐1α.^50^ In addition, HIF‐2α‐dependent CXCL12 secretion in PHD2 (prolyl hydroxylase‐2)‐deficient ECs facilitate PASMCs proliferation and HIF‐2α–selective inhibitor C76 mitigated the established PH rodent models.^51^, ^52^ CXCR4, both HIF‐α target gene and identified hub gene in our study, is a well‐known chemokine receptor for CXCL12. Therefore, it can be assumed that PHD2‐deficient ECs could not only induce PASMCs proliferation, but also foster PASMCs migration and even immune cells accumulation in perivascular spaces in response to CXCL12 release from ECs under hypoxia stress. The modulation of these hub genes would be of great potential for therapeutic benefits in PH as they could impact multiple factors driving PH pathogenesis.

There are some limitations in our study. First, only one dataset of PH lung tissues from human (ie. GSE117261 including 58 PH patients and 25 control subjects) was included. However, the sample size is much larger compared to most of the datasets of human PH lung tissues. Second, the differentially expressed MAGs in hPASMCs in response to PDGF‐BB could not fully represent the genes altered in vivo in PH scenario, and their functional properties in pulmonary vascular remodeling need to be investigated in the future.

**FIGURE 7:**
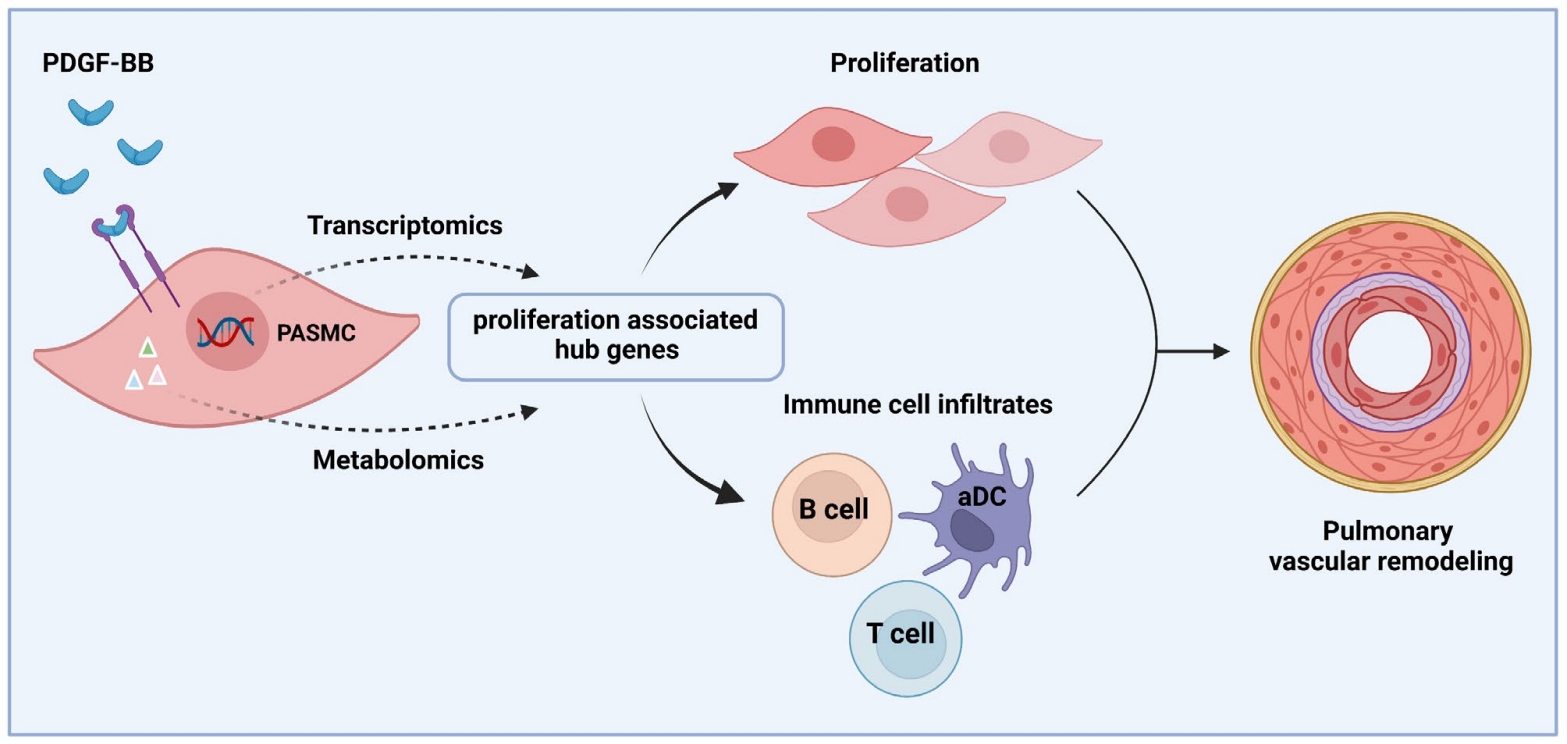
Schematic overview. This schematic overview (created with BioRender.com) illustrates that proliferation associated hub genes in PASMCs in response to PDGF‐BB were explored by virtue of transcriptomics and metabolomics. Those hub genes were demonstrated to be responsible for proliferating PASMCs phenotype and association with immune cell infiltrates into the lung tissues, contributing to the development of pulmonary vascular remodeling

In conclusion, we identified a metabolic/gene profile change in hPASMCs in response to rhPDGF‐BB. The application of multi‐omics allows us to discover hub genes to be responsible for proliferating PASMCs phenotype and to be associated with immune cell infiltrates (Figure 7). We consider that improved molecular understanding of the intricate networks associated with proliferation in PH will have major therapeutic implications.

## CONFLICT OF INTEREST

The authors declare that they have no conflict of interest.

## AUTHOR CONTRIBUTIONS

Y. Yan, Y‐Y. He and Z‐Y. Han designed study design, Y. Yan, R. Jiang and P. Yuan performed data analysis and interpretation, P. Yuan, L. Wen, X‐B. Pang and Z‐C. Jing provided constructive discussion and revised the manuscript. Z‐C. Jing and Y‐Y. He provided funding support. Y. Yan, R. Jiang, Y‐Y. He and Z‐Y. Han wrote the manuscript.

## Supporting information

Fig S1‐3

## Data Availability

The datasets used in the current study are available from the corresponding authors on reasonable request.
