# Supplementary material for: Implication of proliferation gene biomarkers in pulmonary hypertension
Source: Animal Model Exp Med. 2021 Nov 22;4(4):369–80. doi: 10.1002/ame2.12191 (PMC8690983; doi:10.1002/ame2.12191)
Supplement: Supplementary file 1 — Fig S1‐3 [file AME2-4-369-s001.docx]

**Supplementary Materials**

**Implication of** **proliferation gene biomarkers in pulmonary hypertension**

Yi Yan^1,2,#^, Rong Jiang^3,#^, Ping Yuan^3^, Li Wen^4^, Xiao-Bin Pang^5^, Zhi-Cheng Jing^6^, Yang-Yang He^5,^*, Zhi-Yan Han^7,^*

1. Institute for Cardiovascular Prevention (IPEK), Ludwig-Maximilians-University Munich, Munich, Germany;
2. DZHK (German Centre for Cardiovascular Research), partner site Munich Heart Alliance, Munich, Germany;
3. Department of Cardio-pulmonary Circulation, Shanghai Pulmonary Hospital, Tongji University, Shanghai, China;
4. Department of Cardiology, The First Affiliated Hospital, Chongqing Medical University, Chongqing, China;
5. School of Pharmacy, Henan University, Henan, China;
6. State Key Laboratory of Complex, Severe, and Rare Diseases, and Department of Cardiology, Peking Union Medical College Hospital, Chinese Academy of Medical Sciences and Peking Union Medical College, Beijing, China;
7. State Key Laboratory of Cardiovascular Disease and FuWai Hospital, Chinese Academy of Medical Sciences and Peking Union Medical College, Beijing, China

^#^ These authors have contributed equally to this work and share first authorship.

* These authors have contributed equally to this work and share last authorship.

***Correspondence**:

Yang-Yang He, School of Pharmacy, Henan University, North Section of Jinming Avenue, Longting District, Kaifeng, Henan Province, China. E-mail: [hi_heyangyang@163.com](mailto:hi_heyangyang@163.com)

Zhi-Yan Han, State Key Laboratory of Cardiovascular Disease and FuWai Hospital, Chinese Academy of Medical Sciences and Peking Union Medical College, 167 Beilishi Road, Beijing 100037, China. E-mail: [zhiyanhan2006@hotmail.com](http://zhiyanhan2006@hotmail.com)


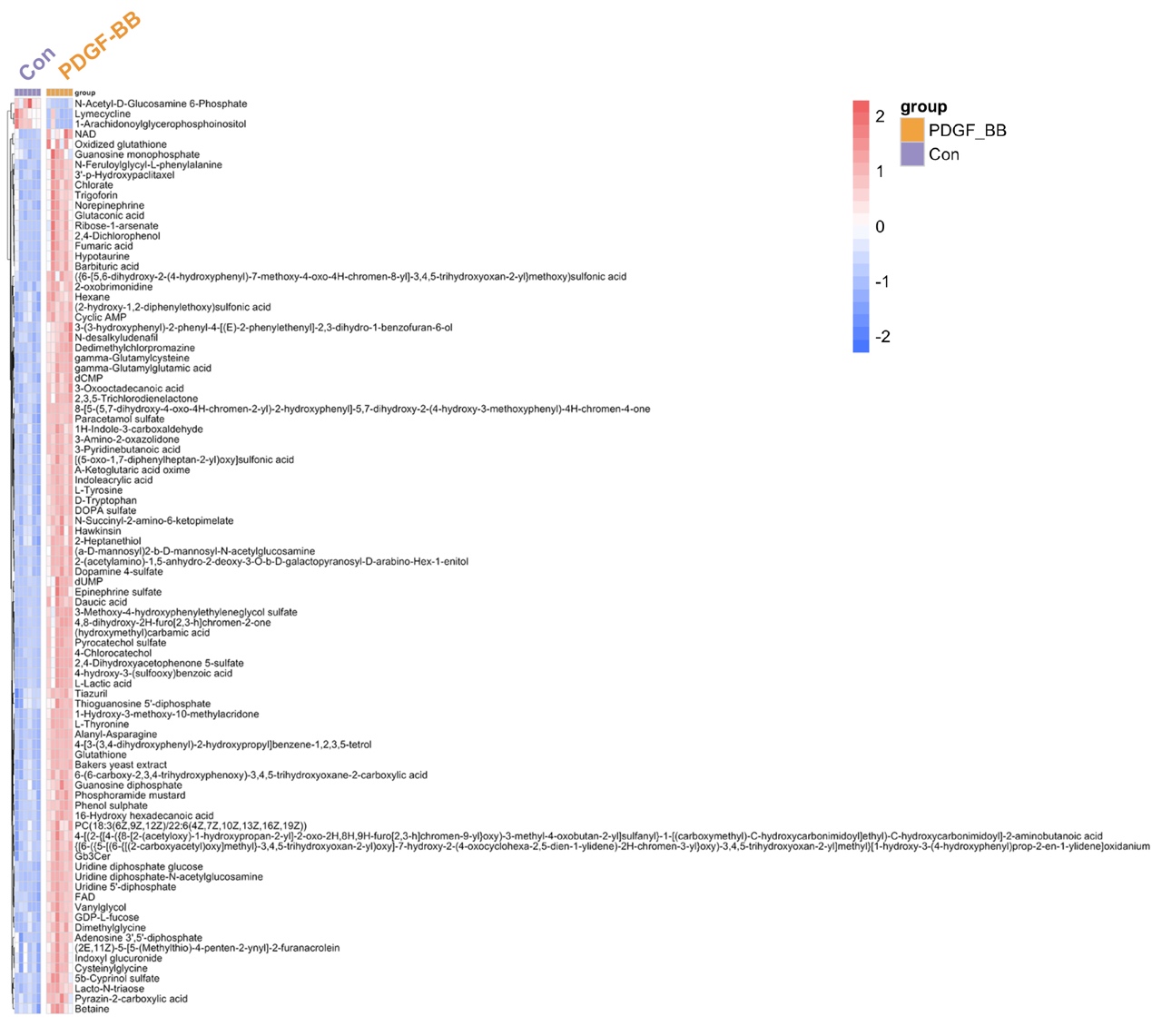


**Supplementary Figure 1. Expression of all distinguishing metabolites in hPASMCs in response to rhPDGF-BB treatment for 24 h.**

Expression of distinguishing metabolites with VIP score > 1, FC > 2 or < 0.5 and P < 0.05 were visualized in heatmap in rhPDGF-BB treated hPASMCs compared to control hPASMCs.

**Supplementary Figure 2. The hPASMCs proliferation in response to rhPDGF-BB.** HPASMCs after rhPDGF-BB (20 ng/mL) treatment had a higher proliferation capacity compared to that of control cells after 24 h. *** *P* < 0.001 as analyzed by Student’s *t* test compared to that of control cells.


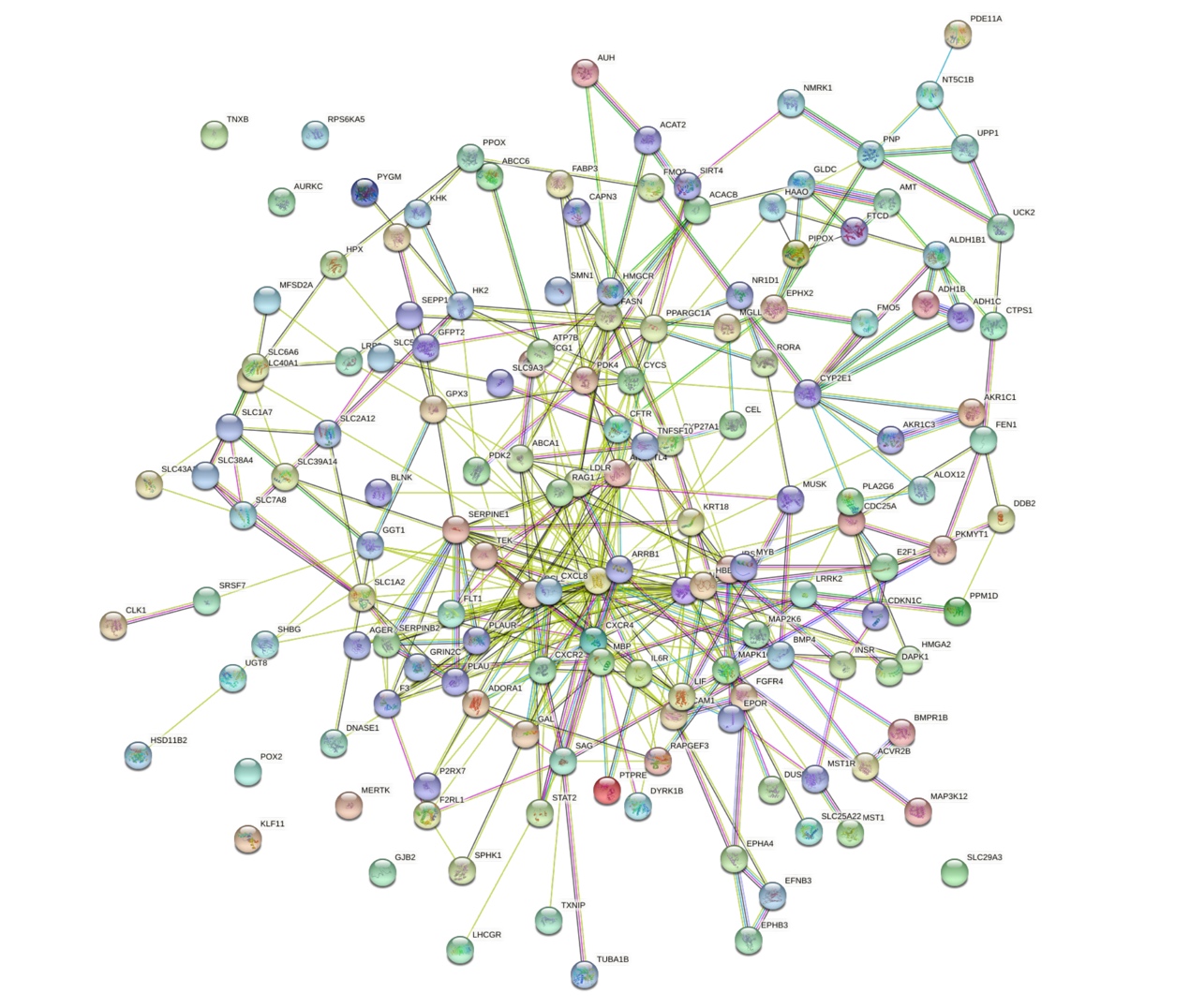


**Supplementary Figure 3. Protein protein interaction of proliferation associated genes in hPASMCs in response to PDGF-BB treatment.**

Protein protein interaction among 152 metabolism associated genes in Figure 3C were analyzed and visualized by online tool STRING (v11.0).
